# Bioactive Properties of the Microwave-Assisted Olive Leaf Extract and Its Incorporation into a Whey Protein Isolate Coating of Semi-Hard Cheese

**DOI:** 10.3390/foods14091496

**Published:** 2025-04-25

**Authors:** Elizabeta Zandona, Maja Vukelić, Karla Hanousek Čiča, Antonio Zandona, Jasna Mrvčić, Maja Katalinić, Ines Cindrić, Almir Abdurramani, Irena Barukčić Jurina

**Affiliations:** 1 Department of Food Technology, Karlovac University of Applied Sciences, Trg J.J. Strossmayera 9, 47000 Karlovac, Croatia; elizabeta.zandona@vuka.hr (E.Z.); ines.cindric@vuka.hr (I.C.); 2 Department of Food Engineering, Faculty of Food Technology and Biotechnology, University of Zagreb, Pierottijeva 6, 10000 Zagreb, Croatia; majavukelic79@gmail.com (M.V.); khanousekcica@pbf.hr (K.H.Č.); jmrvcic@pbf.hr (J.M.); 3 Division of Toxicology, Institute for Medical Research and Occupational Health, Ksaverska Cesta 2, 10000 Zagreb, Croatia; azandona@imi.hr (A.Z.);; 4 Faculty of Food Technology, Josip Juraj Strossmayer University of Osijek, Franje Kuhača 18, 31000 Osijek, Croatia; abdurramanialmir@gmail.com

**Keywords:** biowaste, olive leaves, bioactivities, edible coating, dairy, ripening

## Abstract

The food industry generates large quantities of biowaste, including olive (*Olea europaea* L.) leaves, which are rich in phenolic compounds with proven antioxidant and antimicrobial activity. In this study, a microwave-assisted olive leaf extract was produced and evaluated. Antioxidant potential (20.23 ± 0.31 µmol TE g^−1^), antimicrobial activity against *Staphylococcus aureus* (MIC 17.62 mg GAE g^−1^), and cytotoxic effects in breast (MDA-MB-231 (IC_50_ = 38.9 ± 1.8 µg mL^−1^), MCF-7 (IC_50_ = 58.9 ± 5.4 µg mL^−1^) and prostate cancer PC-3 (IC_50_ = 69.2 ± 7.6 µg mL^−1^) cell models were confirmed. Further, the extract was incorporated into a whey protein isolate (WPI) edible coating mixture and applied to semi-hard cheese over a 60-day ripening period. When applied to cheese, WPI-based coatings enriched with OLE contributed to an improved antioxidant potential (289.79 ± 16.16 µmol TE L^−1^), elevated retention of total phenols and flavonoids, and slightly reduced microbial growth without compromising cheese safety. Compared to the uncoated control, coated samples showed higher total solids (up to 62.87 ± 0.13%, CWPIM) and fat contents (up to 26.59 ± 0.17%, CWPIM), moderated proteolysis (WSN in CWPIM: 3.15 ± 0.09% vs. 4.48 ± 0.02% in C0), maintained cohesiveness and resilience compared to the control, and exhibited less pronounced color deviation (ΔE) in some coated samples during ripening. These results highlight the potential of olive leaf extract as a bioactive, sustainable ingredient for functional edible coatings that improve the nutritional, technological, and microbiological quality of ripened cheese.

## 1. Introduction

Cheese is one of the most widely consumed dairy products globally, valued not only for its sensory properties and cultural diversity but also for its high nutritional content, including proteins, fats, minerals, and bioactive peptides [1,2,3]. During cheese ripening and storage, cheese undergoes significant physicochemical changes, such as variations in moisture, pH, and the development of characteristic flavors and textures [4]. These changes are driven by complex biochemical and microbiological processes involving enzymes and starter cultures. However, these same processes also make cheese susceptible to microbial contamination, which may compromise its quality, safety, and shelf life [5,6].

In recent years, growing consumer demand for minimally processed, preservative-free foods has led to the development of natural preservation strategies. Among these, edible coatings have gained attention due to their ability to reduce moisture loss, limit microbial spoilage, and serve as carriers of bioactive compounds (e.g., plant extracts, essential oils) to enhance antimicrobial and antioxidant properties [7,8,9]. When applied directly to the surface of cheese, such coatings can act as semi-permeable barriers and provide additional bioactivity, potentially through the diffusion of active compounds or interaction with the cheese surface. Numerous studies have explored the application of edible films and coatings in cheese preservation, utilizing materials such as polysaccharides, lipids, and proteins [10]. Despite promising results, challenges remain, related to the functionality, mechanical properties, and bioactive retention in real food matrices. Whey protein isolate (WPI) has become a prominent material for edible films and coatings in cheese preservation due to its unique combination of functional, nutritional, and sustainable properties. As a by-product of the dairy industry, WPI offers biodegradability, transparency, and flexibility while serving as an effective barrier against oxygen, moisture, and microbial contaminants [11,12]. However, challenges persist in optimizing its performance under varying storage conditions and integrating bioactive compounds without compromising mechanical integrity.

In parallel, olive leaf extract (OLE), a by-product of the cultivation of olives and subsequent olive oil production, has attracted attention as a sustainable source of natural antioxidants and antimicrobials, primarily due to its high polyphenol content. Olive leaves are recognized as a rich source of oleuropein, hydroxytyrosol, tyrosol, verbascoside, apigenin, luteolin, rutin, and caffeic and chlorogenic acids [13]. The phenolic profile may vary depending on the cultivar, harvest time, drying method, and extraction technique. Among the available extraction techniques, microwave-assisted extraction (MAE) has gained attention for its efficiency in recovering polyphenols [14]. MAE operates through both thermal and non-thermal effects, enabling the disruption of plant cell walls and enhancing the yield and stability of target compounds. When applied to olive leaves, MAE has been shown to efficiently extract oleuropein and other phenolics while preserving their bioactivity [15]. Multiple studies have highlighted the broad health-promoting effects of olive leaf extract (OLE), encompassing antioxidant, anticarcinogenic, anti-inflammatory, antihypertensive, and cholesterol-lowering activities [16,17]. In addition, the European Food Safety Agency (EFSA) has acknowledged the positive impact of OLE on human health, signaling its potential for use in novel functional foods [18]. Due to its proven bioactivity, OLE has attracted interest not only in pharmaceuticals and supplements but also in food applications, including its incorporation into biodegradable packaging materials such as edible coatings [19]. However, its incorporation into dairy-based coatings and its functionality during cheese ripening remain underexplored.

Therefore, the primary aim of this study was to extract polyphenols from olive leaves using microwave-assisted extraction (MAE) and evaluate its antioxidant, antimicrobial, and anticancer properties. The secondary goal was to incorporate the olive leaf aqueous extract (OLE) into a WPI-based edible coating, and to evaluate its effect on the antioxidant, antimicrobial, and physicochemical properties of semi-hard cheese throughout ripening. By integrating a bioactive plant extract into a dairy-derived edible matrix, this study addresses key challenges in natural cheese preservation and contributes to the development of functional, clean-label packaging solutions.

## 2. Materials and Methods

### 2.1. Production and Characterization of Olive Leaf Extract and WPI-Based Coating

#### 2.1.1. Olive Leaf Extract Production

Olive leaves (*Olea Europaea* L., variety *Oblica*) used for extract production were collected in the Dalmatian region of Croatia during spring (late March). This Mediterranean region is characterized by a warm-summer Mediterranean climate (Csa), with hot, dry summers and mild, wet winters, and predominantly limestone-based soils. Leaves were air dried after harvesting and milled prior to extraction. Olive leaf aqueous extract (OLE) was obtained by microwave-assisted extraction (MAE), according to the previously optimized procedure by Dobrinčić et al. [14] with some modifications. MAE was performed on a Start S Microwave Labstation for Synthesis (Milestone, Bergamo, Italy), using distilled water as the extraction solvent. Extraction parameters were the irradiation time (2 min) and temperature (80 °C). The temperature was controlled via the internal sensor of the system and maintained at 80 °C throughout the extraction. Microwave power was kept constant at 800 W until the target temperature was achieved. After extraction, obtained OLE was centrifugated at 5000 rpm/10 min, and the resulting supernatants were filtered, pasteurized (5 min/90 °C), and cool stored at 4 °C until evaporation. The evaporation was performed on a Heidolph™ Hei-VAP Expert Control ML/G3 rotary evaporator until 16% dry matter remained. The obtained concentrated OLE was freeze stored at −18 °C until further use. Detailed extraction parameters were previously published by Zandona et al. [14], together with the polyphenolic profile of the produced OLE (1.70 mg g^−1^ oleuropein; 0.72 mg g^−1^ rutin; 0.38 mg g^−1^ tyrosol; 0.30 mg g^−1^ caffeic acid; 0.26 mg g^−1^ chlorogenic acid) determined by HPLC analysis.

#### 2.1.2. WPI-Based Coating Preparation

Coating-forming solutions were prepared by slowly dissolving 8% (wt/wt) WPI powder (Impact Whey Isolate, Myprotein^TM^, Manchester, UK) in deionized water. The mixture was heated to 80 °C to denature whey proteins, and temperature was maintained for 20 min with constant stirring. Glycerol was added (5%, wt/wt) as a plasticizer, and the resulting solutions were stirred for approximately 2 h. Then, 10% (wt/wt) sunflower oil (Zvijezda plus d.o.o., Zagreb, Croatia) was incorporated to reduce the water vapor permeability of the coating matrix base, to minimize the dehydration that typically accompanies ripening of cheese [20], and Tween20 (0.2%, wt/wt) was incorporated as an emulsifier to assist in essential oil dissolution via the hydrophilic and hydrophobic parts of that molecule [20]. Both compounds were added under stirring for approximately 20 min at room temperature. Finally, the OLE was added to the mixture. In total, three different samples of edible coatings were prepared. Two coating formulations (WPI75 and WPIM) were prepared using the same volume fraction of olive leaf extract (15% *v*/*v*), but with different total phenol (TP) concentrations, in order to differentiate the effects of phenolic strength. A third coating was prepared without the addition of OLE (WPI0). The TP concentrations of OLE used for WPI coating production were 26.3 mg GAE L^−1^ for WPI75 (the concentration that inhibits 75% of the DPPH radical, according to Zandona et al. [14]) and 15.94 mg GAE mL^−1^ for WPIM (the concentration of the original OLE obtained after MAE and evaporation). The solutions were homogenized at 19,000 rpm for 5 min using an UltraTurrax T25 homogenizer (IKA- Werke GmbH & Co.KG, Staufen, Germany) prior to application. The final TP concentrations in WPI75 and WPIM coatings were 3.95 µg mL^−1^ and 2.39 mg mL^−1^, respectively.

#### 2.1.3. Antimicrobial Activity of Olive Leaf Extract and WPI-Based Coating

In the first step, the antimicrobial activities of the extract or coating were determined using the disc diffusion method to verify the efficacy of the extract on all tested strains. To evaluate the antimicrobial properties of the extract, thirteen microorganisms were used for this study: Gram-positive bacteria (*Staphylococcus aureus*, *Bacillus subtilis*, *Enterococcus faecium*, *Listeria monocytogenes*), Gram-negative bacteria (*Pseudomonas aeruginosa*, *Escherichia coli*, *Salmonella enterica s. Typhimurium*), lactic acid bacteria (*Leuconostoc mesenteroides*, *Lactobacillus plantarum*), and yeasts (*Candida albicans*, *Candida utilis*, *Rhodotorula* sp., and *Saccharomyces cerevisiae*). The microorganisms were stored on slant agar in the microorganism collection of the Laboratory of General Microbiology and Food Microbiology and the Laboratory of Fermentation and Yeast Technology of the Faculty of Food Technology and Biotechnology, University of Zagreb (Croatia). After overnight growth of cultures under anaerobic conditions at 37 °C in Mueller Hinton broth (Gram-positive and Gram-negative bacteria), MRS broth at 32 °C (lactic acid bacteria), and Mueller Hinton broth with 2% glucose at 28 °C (yeast), cell density was adjusted to 10^6^ cell mL^−1^ [21]. Agar plates were then inoculated directly from the suspension with a sterile cotton swab according to the CLSI protocol [21]. Filter paper discs (Macherey-Nagel, Duren, Germany) were then placed on the inoculated medium using flamed forceps, and extract (10 µL; 20 µL) was applied to the discs (diameter = 6 mm). The coatings with the extract were shaped into circles with a diameter of 6 mm and applied directly to the agar. Kanamycin (50 µg) and nystatin (100 U) (BioLab Inc., Budapest, Hungary) were used as positive controls. Petri dishes were incubated, and zones of inhibition (mm) were measured with a ruler after 24 h.

The minimum inhibitory concentration (MIC) was determined by staining with resazurin (0.001%, 50 µL). Briefly, Mueller Hinton broth was used for the growth of *S. aureus*. A fresh 24 h broth culture was used, and the final density was set at 10^6^ CFUmL^−1^. Two-fold dilution series of the extract as well as kanamycin were prepared in microtiter plates with a total volume of 200 µL. The microtiter plate was incubated at 37 °C for 24 h, after which the cells were stained with resazurin. The minimum concentration of the test substance that completely inhibits bacterial growth (the change in blue color to pink was not observed) is referred to as the MIC. To determine the number of live cells in an MIC microtiter well, serial tenfold dilution was performed in sterile saline and aseptically spread on Mueller Hinton agar plates. All agar plates were incubated at 37 °C for 24 h, and colonies were counted to determine CFUmL^−1^.

#### 2.1.4. Total Phenol, Total Flavonoid Content, and Antioxidant Activity of Olive Leaf Extract

The total phenol content (TPC) was determined by the Folin–Ciocalteu method, modified according to Shortle et al. [22]. The standard curve (y = 0.0031x) was plotted using 500 mg L^−1^ gallic acid (GA, Sigma-Aldrich, St. Louis, MO, USA) as a stock solution. The total flavonoid (TFC) content was determined according to Aryal et al. [23] with slight modifications. The standard curve (y = 0.0098x) was derived using 2 mmol L^−1^ quercetin (QC, Sigma-Aldrich, St. Louis, MO, USA) as a stock solution. The antioxidant activity (AA) of WPI-based coating samples was determined by the ferric reducing antioxidant power (FRAP) method according to Benzie et al. [24]. The standard curve (y = 0.0014x) was plotted using 2 mmol L^−1^ Trolox (Sigma-Aldrich, St. Louis, MO, USA) as a stock solution.

#### 2.1.5. Cytotoxicity and Activation of Caspases

The triple-negative breast cancer cells MDA-MB-231, ER-positive breast cancer cells MCF-7, and prostate cancer cells PC-3 were used for cell-based assays. Cells were a gift from colleagues from the Division of Molecular Medicine, Ruđer Bošković Institute, Zagreb, and were grown in Dulbecco’s Modified Eagle Medium (Sigma-Aldrich, Steinheim, Germany) supplemented with 10% fetal bovine serum (Sigma-Aldrich, Steinheim, Germany) and 1% Penicillin/Streptomycin (Sigma-Aldrich, Steinheim, Germany) at 37 °C in a 5% CO_2_ atmosphere in a HeraCell Vios 160i incubator (Thermo Fisher Scientific, MA, USA), and the medium was changed every 2–3 days.

Cytotoxicity was determined based on the succinate dehydrogenase mitochondrial activity using the CellTiter 96^®^ AQueous One Solution Cell Proliferation Assay (Promega, Madison, WI, USA), which is directly proportional to the number of metabolically active cells, as described previously [25]. We seeded 20,000 cells per well in 96-well plates and exposed those for 24 h to OLE (water extract) in the concentration range up to 500 mg L^−1^. After the incubation, we measured the absorbance at 492 nm on the InfiniteM200PRO plate reader (Tecan Austria GmbH, Salzburg, Austria). Data were taken from at least two independent experiments (each treatment performed in duplicate) and plotted as a percentage of cytotoxicity compared to the control, untreated cells.

Activation of specific caspases was determined based on the substrate and fluorogenic indicators from a Caspase-3, Caspase-8, and Caspase-9 Multiplex Activity Assay Kit (Abcam, Cambridge, UK). The procedure followed a previously described protocol [26]. Cells were seeded at a density of 20,000 cells/well in 96-well black plates and exposed for 6 h to OLE in concentrations corresponding to its IC_25_, IC_50_, and IC_75_ concentrations determined in a 24 h exposure experiment. After the incubation, 100 μL of caspase assay loading solution (Abcam, Cambridge, UK) was added to each well and incubated in the dark for 30–60 min, and fluorescence (Caspase-3 at 535 nm and 620 nm; Caspase-8 at 490 nm and 525 nm; Caspase-9 at 370 nm and 450 nm) was measured on an InfiniteM200PRO plate reader. A total of 3 µmol L^−1^ staurosporine (SS, Sigma-Aldrich, Germany) was used as a positive control. Data were taken from at least two independent experiments (each treatment performed in duplicate) and presented as a normalized relative fluorescence signal to the control, untreated cells.

### 2.2. Production of Cheese Samples with WPI-Based Coating

For cheese production, 12 L of fresh pasteurized and non-homogenized milk with 3.2% milk fat, 4.8% carbohydrates, and 3.3% proteins (Veronika mini dairy, Desinić, Croatia) was used. Fresh milk was heated to 35 °C, after which 0.03% (w/v) of CaCl_2_ (Gram-mol, Croatia), previously dissolved in a warm milk, and 1% (*v*/*v*) of Choozit^®^ Probat 222 LYO 100 DCU (IFF Danisco, Germany) were added. Finally, 0.02% (*v*/*v*) of rennet (BioRen^®^ Classic 80LHA150 liquid rennet) dissolved in 10 times the amount of distilled water was added. The milk was incubated at 37 °C for 1 h. The resulting curd was cut into cubes and left some time to separate the whey. After the whey was separated, the curd was transferred into small molds covered with cheesecloth. All the cheese curds were pressed by their own weight for approximately 2–4 h and then subjected to a 5 kg weight for 24 h with occasional turning. After pressing, the cheeses were removed from the mold and placed into previously prepared brine (18% NaCl solution with 0.15% CaCl_2_) for approximately 3 h, after which they were dried. Whey-based coating mixtures were applied to both sides of the cheeses by manually spreading the solution over the surface, and each side was subsequently dried for approximately 12 h. In total, four different cheese samples were produced: control cheese (C0), cheese coated in WPI-based coating without the addition of OLE (CWPI0), cheese coated in WPI-based coating with the addition of OLE (TP concentration = 3.95 µg mL^−1^) (CWPI75), and cheese coated in WPI-based coating with the addition of OLE (TP concentration = 2.39 mg mL^−1^) (CWPM). All cheese samples were then subjected to a ripening process at 11–13 °C and 75–85% humidity for 60 days. The samples were taken for physico-chemical and microbiological analyses at days 1, 15, 30, 45, and 60.

#### 2.2.1. Physico-Chemical Analyses of Cheese

The total solids of cheese was determined by drying the samples at 102 ± 2 °C until they reached a constant mass [27]. The ash content was determined gravimetrically by drying at 550 °C until completely ashed [28].

The active acidity (pH) of cheese was determined by a ProfiLine pH 3110 pH meter (Xylem Analytics, Weilheim in Oberbayern, Germany). Titratable acidity (°SH) was determined according to the AOAC standard method, which includes mixing grated cheese with warm water (40 °C), filtering and titrating with 0.1 M NaOH, and using phenolphthalein as the indicator [29]. Milk fat content was determined by a butyrometric method according to ISO 3432:2008 [27]. The water activity (a_w_) of cheese was determined by a HygroPalm HP23 aw meter (Rotronic, Bassersdorf, Switzerland). To monitor proteolytic changes in cheese samples, total nitrogen (TN), trichloroacetic acid-soluble nitrogen (TCA), and water-soluble nitrogen (WSN) were determined by the Kjeldahl method according to the AOAC 991.21-991.23 methods [30].

The color of cheese samples was determined according to the CIElab system using a CM-CM-700d colorimeter (Konica Minolta, Tokyo, Japan) with a D65 light source, as previously described in the study by [15,31], and the L*, a*, and b* parameters were measured and the total color difference (ΔE*) calculated following Equation (1):

(1) $$∆E*=\sqrt{{\left(L*-{L*}_{ref}\right)}^{2}+{\left(a*-{a*}_{ref}\right)}^{2}+{\left(b*-{b*}_{ref}\right)}^{2}}$$

where L*, a*, and b* refer to the test samples and L*ref, a*ref, and b*ref to the control samples. The obtained results were interpreted according to the model described by Mokrzycki and Tatol [32] (Table 1).

The texture of cheese curds was determined using a texture analyzer (Ametek Lloyd Instruments Ltd., West Sussex, UK) as Marušić Radovčić et al. [33] previously described. Cheese samples were twice compressed up to 50% deformation with a 50 kg cell, at a speed of 1 m s^−1^ with a 5 s interval between two cycles. The obtained data were processed using NexygenPlus software version 3.0 which generated values for adhesiveness (Nmm), adhesive force (N*), cohesiveness, hardness (N), gumminess (N), chewiness (Nmm), stringiness (mm), resilience, fracture (N), and springiness (mm).

#### 2.2.2. Microbiological Analyses of Cheese

Microbiological analyses of cheese were performed by using a plate pouring method. Cheese samples were prepared by dissolving 20 g of cheese in 180 mL of sterile 2% sodium citrate solution tempered to 45 °C (ISO 6887-5:2010, [34]). The obtained suspension was used for the preparation of further dilutions. The total viable cell count was enumerated in Tryptic Glucose Yeast Agar (30 °C/48–72 h), *Enterobacteriaceae* in Violet Red Bile Glucose Agar (37 °C/24 h), coagulase-positive *Staphylococci* in Baird Parker Agar with added Egg Yolk emulsion (37 °C/24–48 h), and yeasts and molds in Sabouraud Dextrose Agar CAF 50 (room temperature/72 h) (all Biolife, Milan, Italy). Also, the samples were analyzed for the presence of *E. coli* (ISO 16649-2, 2001 [34]), *Salmonella* sp. (ISO 6579-1, 2017 [35]), and *L. monocytogenes* (ISO 11290-2, 2017 [36]). The obtained results were compared to microbiological criteria for semi-hard cheeses produced from pasteurized milk set by Commission regulation (EC) 2073/2005 [37] and by the National Guide [38].

#### 2.2.3. Total Phenol, Total Flavonoid Content, and Antioxidant Activity in Cheese

Cheese samples were prepared according to Apostolidis et al. [39] by homogenizing 20 g of cheese with 20 mL of distilled water and centrifuging the prepared suspension at 10,000 rpm for 10 min at 5 °C. The obtained supernatant was separated and stored at 4 °C until further analysis of the total phenol content, total flavonoid content, and antioxidant activity (according to methods described in Section 2.1.3. Total phenol, total flavonoid content, and antioxidant activity of OLE).

### 2.3. Statistical Analysis

Each experiment was repeated in triplicate, and the obtained results were expressed as mean values ± standard deviations (SD). Two-way or one-way ANOVA using a multiple-comparison Dunnett’s test was performed to determine the significant effect of factors (a—sample C0 vs. samples, b—sample CWPI0 vs. samples, and c—specific sample on day 1 vs. days, *p* ≤ 0.05)), except for ash, total solids, and fat content (%), where an unpaired *t*-test (*c*—specific sample on day 1 vs. day 60, *p* ≤ 0.05) was used. Prior to applying ANOVA, where applicable, data were tested for normality and homogeneity of variance. Only datasets that met these assumptions were subjected to ANOVA. Cytotoxicity and caspase activities are presented as means ± standard error (SE) and statistical significance is set as follows: & *p* ≤ 0.05; # *p* ≤ 0.01; $ *p* ≤ 0.001; and * *p* ≤ 0.0001. All statistical analyses and graphs were run on the GraphPad Prism software version 9 (San Diego, CA, USA).

## 3. Results and Discussion

### 3.1. Antioxidant, Antimicrobial, and Cytotoxic Effects of Olive Leaf Extract

The TPC and TFC contents were 35.25 ± 0.65 mg GAE g^−1^ and 3.63 ± 0.10 mg QE g^−1^, respectively. AA of OLE was determined by the FRAP method, and capacity was 38.96 ± 1.27 mg TE g^−1^ (GAE—gallic acid equivalent; QE—quercetin equivalent; TE—Trolox equivalent).

The antimicrobial activity of the extract was tested against Gram-positive and Gram-negative bacteria, lactic acid bacteria, and yeasts using the disk diffusion method, the microdilution method, and the colony counting method. Of the thirteen microorganisms used, only the growth of *S. aureus* was inhibited. The antimicrobial activity was significantly weaker than that of the antibiotic kanamycin, which was used as a positive control (Figure S1). Further, microdilution methods were performed to determine the minimum inhibitory concentrations, and the colony counting method was used to further characterize the antimicrobial activity of the extract. The results showed that the number of *S. aureus* cells growing both in the presence of 50% extract and in the presence of 10 µg mL^−1^ of kanamycin was inhibited, and the number after 18 h was equal to the original inoculum of 10^6^ cells, while the number of cells in undisturbed cultivation was 10^9^ (Figure S2). The determined MIC for *S. aureus* was 17.63 mg GAE g^−1^. Besides *Salmonella* spp., *Escherichia coli,* and *Listeria monocytogenes*, *Staphylococcus aureus* is one of the most common causes of contamination of pasteurized and raw milk cheeses, which poses a greater risk for the growth and survival of pathogenic bacteria due to their high moisture, pH, and salt content. Once again, we confirmed that OLE really has an antimicrobial effect (and mostly against pathogens like *S. aureus*) [40]. The exact antimicrobial mechanism of polyphenols from olive leaf extract is not known, but it has been reported that oleuropein contained in olive fruits and leaves inhibits the growth rate of several microorganisms [41].

**Figure 1:**
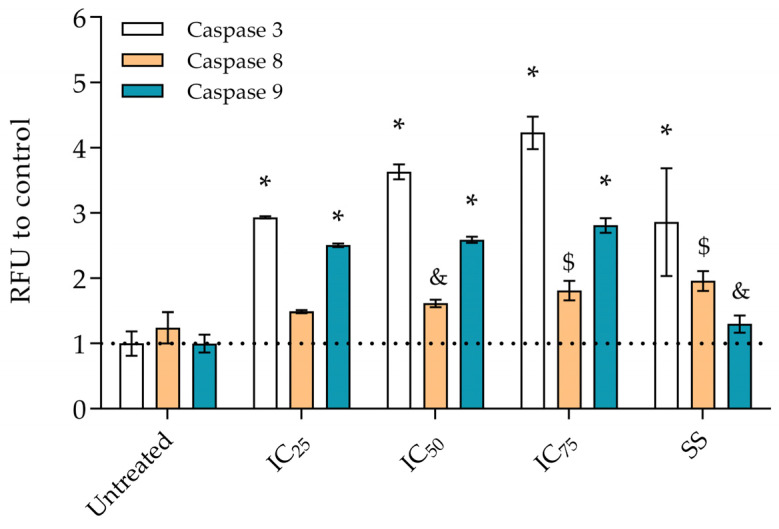
Specific caspase activity: caspase 8 (orange), caspase 9 (turquoise), and caspase 3 (white) in MDA-MB-231 cells after 6 h exposure to IC_25_ (30.2 µg mL^−1^), IC_50_ (38.9 µg mL^−1^), and IC_75_ (48.9 µg mL^−1^) concentrations of OLE. Staurosporine (SS, 3 µmol L^−1^) was used as a positive control. Results are presented as relative fluorescence units (RFU) to untreated cells. Statistical significance: Ordinary one-way ANOVA—Dunnett’s test (& *p* < 0.05; $ *p* < 0.001; * *p* < 0.0001).

OLE was cytotoxic to breast cancer cells MDA-MB-231 (IC_50_ = 38.9 ± 1.8 µg mL^−1^), MCF-7 (IC_50_ = 58.9 ± 5.4 µg mL^−1^), and prostate cancer cells PC-3 (IC_50_ = 69.2 ± 7.6 µg mL^−1^) after 24 h treatment. The highest anticancer effect was observed on the most progressive cancer type, triple-negative MDA-MB-231 cells. To evaluate whether in these MDA-MB-231 cells OLE activates apoptosis, as a preferred type of cell death in cancer treatment, the activity of three specific caspases was monitored after OLE exposure, and the results are presented in Figure 1. Both initiator caspases 8 and 9 were activated by OLE and consequently executioner caspase 3, indicating that the activation of apoptosis is a desirable anticancer property [42].

Additionally, when present in the functional food, polyphenol compounds could help in the prevention and treatment of many cancers [43]. Therefore, the cytotoxicity profiling of plant extracts is not only important for assessing and confirming the safety of their use for traditional purposes but also for paving the way in the discovery of new active compounds. Likewise, we have shown that OLE has an anticancer effect on several of the most common and progressive cancer types. Interestingly, OLE was the most effective on the most resistant MDA-MB-231 cancer cells, compared to other plant extracts, where these cells were the least sensitive [44]. Also, OLE extract proved to be effective in the activation of apoptosis (through the activation of the caspase 3 signaling cascade). A similar effect was observed for *Phoenix dactylifera* L. extract [45], but at a 2-fold lower concentration than OLE. Further, polyphenols, as components of such extracts, have the ability to modulate cell signaling pathways and inhibit tumor growth, which supports their potential as key active components for anticancer activity [43,46].

### 3.2. Physico-Chemical Properties of the Cheese with WPI Coating

Throughout 60 days of ripening, all cheese samples exhibited characteristic changes in physicochemical properties, with observable differences between control (C0) and coated samples (CWPI0, CWPI75, CWPIM; Table 2). The application of WPI-based coatings, especially those enriched with OLE, influenced moisture retention, proteolysis, color, and texture development.

Water activity (a_w_) decreased slightly over time across all samples, with minimal differences among treatments. Lactic acid content increased during ripening and decreased the pH value. These results are in accordance with Kilic and Koyuncu [47]. CWPIM showed the lowest lactic acid concentration at the end of the ripening (2.68 ± 0.03%), indicating that coatings might have modulated acid production or retention.

The total solid content increased in all samples during ripening, which is typical for semi-hard cheeses due to progressive moisture loss, except in CWPIM, where a significant decrease was determined (*p* ≤ 0.05). The ash content, representing the mineral fraction, showed a marked decrease from day 1 to day 60 in all samples, most likely due to changes in salt diffusion and matrix solubility. The fat content generally increased during ripening, reflecting moisture loss and concentration effects. On day 60, the highest fat content was observed in CWPIM (26.59 ± 0.17%), which was statistically significant compared to C0 (25.24 ± 0.18%) and CWPI0 (25.54 ± 0.06%) (Table 3). This suggests that the WPI coatings enriched with OLE may have contributed to reduced moisture loss, thus increasing the fat percentage on a dry matter basis.

The protein content decreased over time in all samples, which is consistent with the expected proteolytic degradation during ripening (Table 4). However, coated samples (CWPI75 and CWPIM) retained a higher protein content at day 60 (19.02 ± 0.12 and 19.35 ± 0.05%, respectively) compared to the control (14.75 ± 0.07%) (*p* ≤ 0.05), indicating a potential protective effect of coatings against excessive protein breakdown or leaching. Proteolysis progressed with ripening, as reflected in increasing levels of both TCA-soluble nitrogen (TCA-SN) and water-soluble nitrogen (WSN; Table 4). The highest WSN values were recorded in the control (C0) at day 60 (4.48 ± 0.02%), while the lowest were in CWPIM (3.15 ± 0.09%). The difference between C0 and coated samples, especially CWPI75 and CWPIM, was significant (*p* ≤ 0.05), suggesting that WPI coatings with OLE slowed proteolytic activity. Similarly, the TCA-SN content was lower in CWPIM than in the control (C0).

According to the results of color measurements (Table 5) with sample C0 as a reference, a clear difference in color was visible (∆E* values greater than 3.0) in all cheese samples (CWPI0, CWPI75, and CWPIM) during the ripening period, and the observer could note two different colors after 60 days (∆E* values greater than 5.0).

The evolution of cheese texture over 60 days of ripening is summarized in Table 6. All measured textural parameters—hardness, gumminess, cohesiveness, elasticity, and resilience—were significantly influenced by ripening time and the application of WPI-based coatings, particularly those enriched with OLE. Hardness increased progressively across all samples, reflecting moisture loss and protein network tightening typically observed in semi-hard cheeses. C0 was significantly softer (*p* < 0.05) than coated cheeses CWPI0, CWPI75, and CWPIM, which is in accordance with results previously published by Ramos et al. [20]. Cohesiveness gradually declined with ripening, but coated samples maintained higher cohesiveness, implying better preservation of internal structural integrity. This may be due to reduced enzymatic degradation within the matrix, possibly mediated by antioxidant components in the coating. Resilience remained higher in coated samples compared to the control (C0), indicating better recovery ability upon deformation.

### 3.3. Total Phenols, Flavonoid Content, and Antioxidant Activity in Coated Cheese

**Figure 2:**
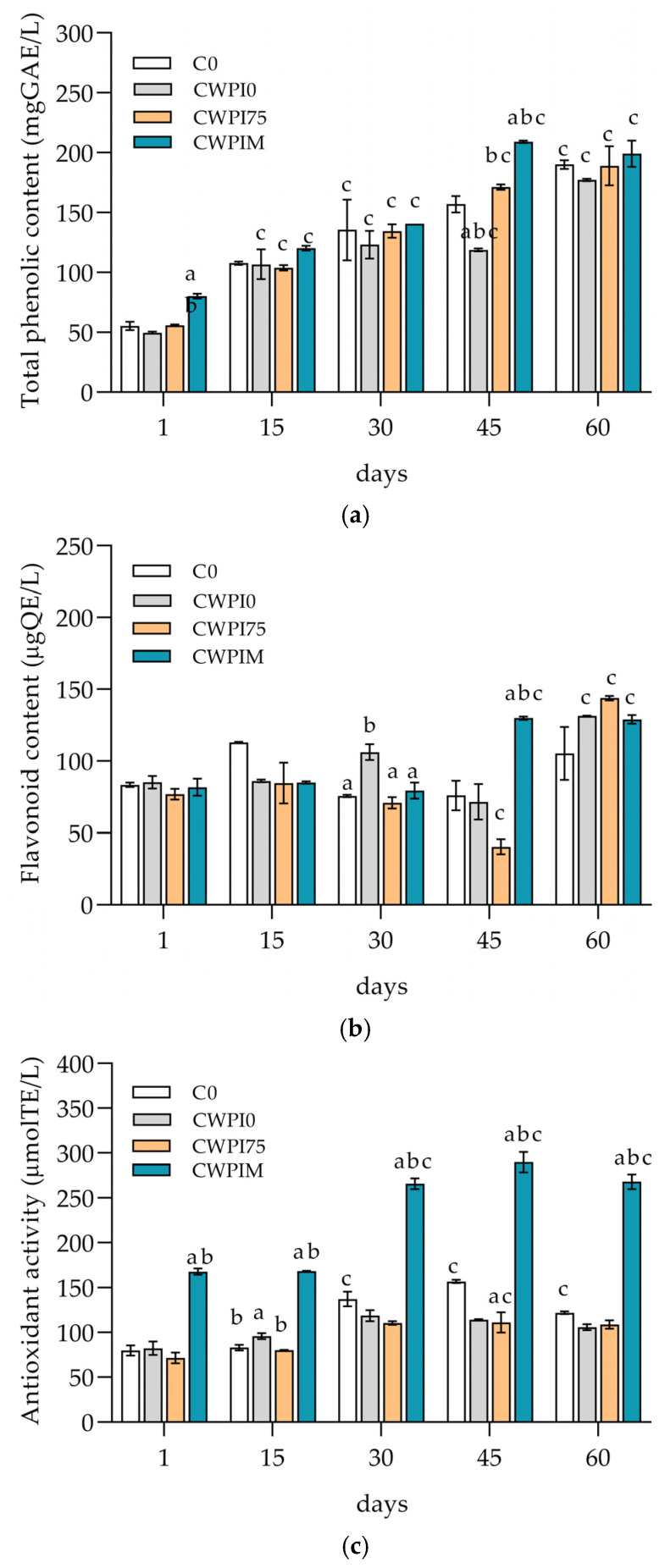
Total phenol content expressed as gallic acid equivalents (mg GAE L^−1^) (**a**), total flavonoid content expressed as quercetin equivalents (µg QE L^−1^) (**b**), and antioxidant activity determined by the FRAP method and expressed as Trolox equivalents (µmol TE L^−1^) (**c**) in the control cheese (C0) and samples with WPI-based coatings (CWPI0, CWPI75, and CWPIM) during ripening. Statistical significance: two-way ANOVA—Dunnett’s test (a control C0 vs. all samples, b sample CWPI0 vs. all samples, and c specific sample on day 1 vs. other days, *p* ≤ 0.05).

The evolution of the total phenolic content (TPC), total flavonoid content (TFC), and antioxidant capacity (FRAP) during 60 days of ripening is shown in Figure 2a,b. All three parameters were influenced by the ripening time and the presence of WPI coatings, with or without added OLE. TPC increased significantly across all samples, with initial values ranging from 49.66 ± 1.22 mg GAE L^−1^ (CWPI0 at 1st day of ripening) to 209.14 ± 1.46 mg GAE L^−1^ (CWPIM at 45th day of ripening (*p* ≤ 0.05). Kilic and Koyuncu also reported the same effect in kashar cheese with the addition of fruit powder [47]. The increase was attributed to moisture loss, concentration effects, and the presence of phenolic compounds from OLE. Coated samples, particularly CWPIM, showed slightly elevated TPC values compared to the control (C0), especially in the later stages of ripening. OLE is rich in polyphenolic compounds, which are most abundant and have been given enormous attention due to their healthful properties [48]. It has been proven that a higher percentage of polyphenols contributes to bioactivity [49], which was also observed in this study in the case of sample CWPIM, which had the highest proportion of OLE and therefore TPC. Thus, it was shown that the addition of OLE can significantly improve the bioactivity of the cheese itself.

Fluctuations in TFC were observed, with a general increasing trend. Although differences between treatments were minimal at day 1, CWPIM exhibited a significantly higher TFC at day 45 compared to CWPI0 and CWPI75 (*p* ≤ 0.05). Nevertheless, by day 60, all samples showed an elevated flavonoid content (around 135 µg QE L^−1^), with no statistically significant differences, suggesting that the ripening time was the main determinant of TFC, rather than the coatings themselves.

The AA, determined by the FRAP method, showed a markedly different pattern (Figure 2c). CWPIM consistently exhibited the highest antioxidant activity throughout ripening, reaching 265.86 ± 8.59 and 289.79 ± 16.16 µmol TE L^−1^ at days 30 and 45, respectively (*p* ≤ 0.05), for both the control and other coating treatment. CWPI75, on the other hand, showed significantly low antioxidant activity among all samples during most of the ripening period, while CWPI0 and C0 remained comparable. These results indicate that the presence of OLE in the concentrated form (CWPIM) was particularly effective in enhancing the antioxidant potential of the cheese matrix, supporting the functional role of phenolic-rich coatings in preserving oxidative stability during ripening. However, the antioxidant capacity of extracts can be affected by temperature, light, and the pH value [50].

### 3.4. Cheese Microbiology and Antimicrobial Activity of WPI-Based Coating

**Figure 3:**
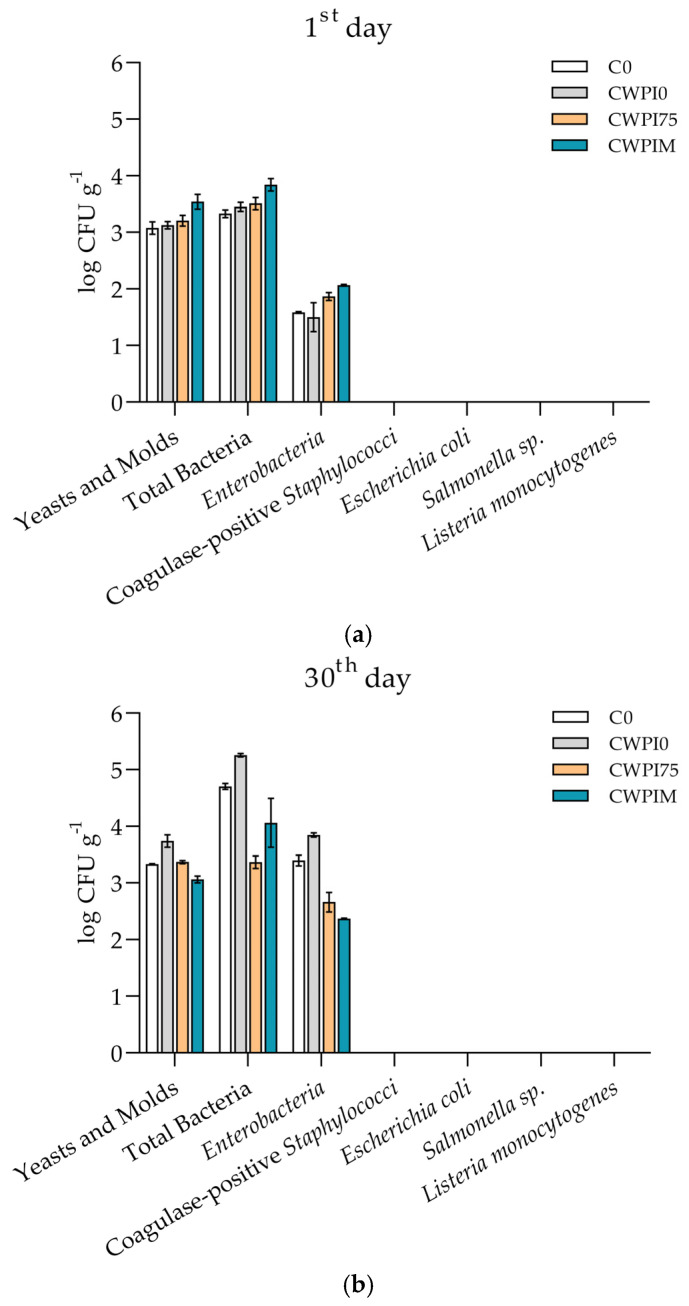

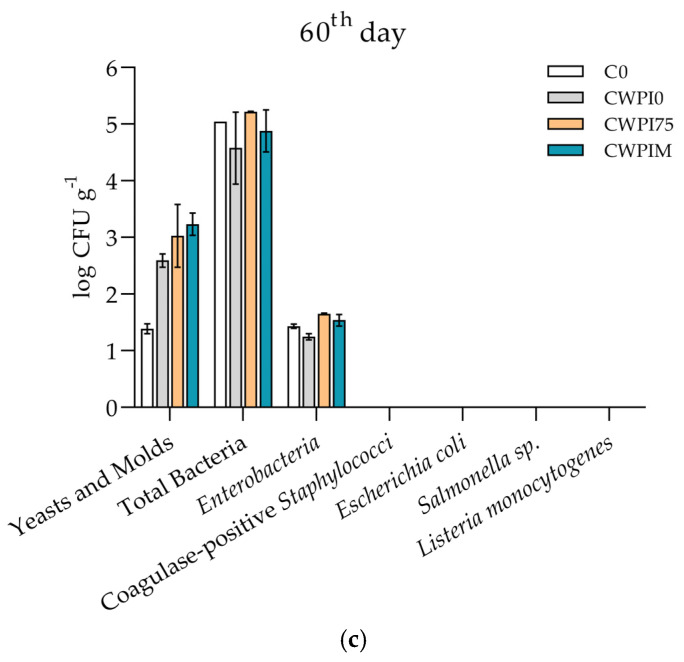
Number of microorganisms (log CFU g^−1^) in the control cheese (C0) and samples with WPI-based coatings (CWPI0, CWPI75, and CWPIM) on the 1st (**a**), 30th (**b**), and 60th days (**c**). There was no statistical significance between samples (one-way ANOVA—Dunnett’s test, *p* ≤ 0.05).

The microbiological quality of the control (C0) and coated cheese samples (CWPI0, CWPI75, and CWPIM) was evaluated on days 1, 30, and 60 of ripening (Figure 3). Total aerobic mesophilic bacterial counts across all samples ranged between 4.5 and 5.0 log CFU/g, remaining relatively stable throughout the ripening period. Slight reductions in total aerobic mesophilic bacteria and yeasts/molds were observed in CWPI75 and CWPIM compared to the control, particularly at later stages, although differences were not statistically significant (*p* > 0.05). *Enterobacteria* and coagulase-positive *Staphylococci* were detected at moderate levels (3.0–3.5 log CFU/g) at day 1, with a gradual decline noted in samples coated with OLE (CWPI75 and CWPIM), suggesting a potential mild antimicrobial effect of the bioactive components. Importantly, *Escherichia coli*, *Salmonella* spp., and *Listeria monocytogenes* were not detected (absence in 25 g) in any sample at any time point. These findings are in full compliance with the microbiological criteria set by Commission Regulation (EC) No. 2073/2005 for cheeses made from pasteurized milk [27], as well as with national guidelines [28]. Overall, the application of WPI-based coatings enriched with OLE did not compromise the microbiological safety of the cheeses and did not inhibit lactic acid bacteria for at least 60 days, and a similar effect was reported by Ramos et al. [20]. On the contrary, they may offer a modest protective effect by inhibiting the growth of spoilage or opportunistic microorganisms during ripening, supporting their use as functional edible coatings in cheese preservation.

In addition to microbial enumeration in cheese, the antimicrobial potential of the WPI-based coatings was evaluated in vitro against a panel of Gram-positive and Gram-negative bacteria, lactic acid bacteria, and yeasts. Out of thirteen microorganisms used, only the growth of *S. aureus* was inhibited by sample WPIM, with the inhibition zone ranging from 8 to 12 mm (Figure S3). No inhibition zones were observed for the other two samples (WPI0 and WPI75) (Figure S3), suggesting that the antimicrobial activity was attributable to the higher concentration of OLE in the WPIM formulation. Still, antimicrobial activity was significantly weaker than that of the positive control (kanamycin), indicating limited bactericidal potency under the tested conditions. These findings confirm that the previously established antioxidant and antimicrobial properties of OLE were at least partially retained following its incorporation into the WPI-based coating matrix. Although the coating showed only mild antimicrobial effects in vitro, its main benefit in situ may be in maintaining antioxidant activity and helping to keep microbial growth under control during ripening.

## 4. Conclusions

This study demonstrated that the incorporation of olive leaf extract (OLE) into whey protein isolate (WPI)-based edible coatings can enhance the functional and technological quality of semi-hard cheese during ripening. The applied coatings contributed to improved moisture retention, moderated proteolysis, and influenced the textural and color attributes of cheese. Notably, samples coated with WPI enriched with concentrated OLE (CWPIM) exhibited the highest antioxidant capacity, increased the retention of total phenols and flavonoids, and slightly reduced microbial counts, without compromising microbiological safety. Although the in vitro antimicrobial activity of the coatings was limited—showing an inhibitory effect only against *S. aureus*—the presence of OLE contributed to overall microbial stability during cheese ripening. Physicochemical analysis further indicated that WPI-based coatings may act as a protective barrier, reducing moisture and protein loss and enhancing structural integrity. These findings support the use of olive-derived bioactive compounds and dairy by-products as sustainable functional ingredients, offering a promising strategy for improving the shelf life, nutritional value, and oxidative stability of cheese, while contributing to circular economy principles.

## Figures and Tables

**Table 1:** Typical scale of the total color difference for understanding human visual perception [32].

ΔE* Value	Observer Color Difference Perception
0–1.0	Observer does not notice the difference
1.1–2.0	Only an experienced observer can perceive the difference
2.1–3.5	Unexperienced observer also notices the color difference
3.6–5.0	A clear distinction in color is visible
>5.0	Observer notices two different colors

**Table 2:** Water activity (a_w_), lactic acid content (%), and pH values of control (C0) and WPI-coated cheese samples (CWPI0, CWPI75, and CWPIM) during 60 days of ripening.

Day	Sample	a_w_	Lactic Acid (%)	pH
1	C0	0.92 ± 0.06	0.74 ± 0.08	6.66 ± 0.12
	CWPI0	0.92 ± 0.08	0.63 ± 0.08	6.65 ± 0.19
	CWPI75	0.92 ± 0.07	0.67 ± 0.08	6.62 ± 0.11
	CWPIM	0.92 ± 0.18	0.72 ± 0.08	6.57 ± 0.21
15	C0	0.94 ± 0.19	2.22 ± 0.02 ^bc^	5.82 ± 0.15 ^c^
	CWPI0	0.92 ± 0.13	2.43 ± 0.05 ^ac^	5.86 ± 0.03 ^c^
	CWPI75	0.94 ± 0.17	2.32 ± 0.10 ^c^	6.03 ± 0.19 ^c^
	CWPIM	0.93 ± 0.03	2.41 ± 0.08 ^ac^	5.70 ± 0.16 ^c^
30	C0	0.90 ± 0.15	2.90 ± 0.07 ^bc^	5.59 ± 0.02 ^bc^
	CWPI0	0.89 ± 0.01	3.12 ± 0.03 ^ac^	5.31 ± 0.11 ^ac^
	CWPI75	0.89 ± 0.04	3.11 ± 0.09 ^ac^	5.49 ± 0.11 ^c^
	CWPIM	0.90 ± 0.14	3.10 ± 0.05 ^ac^	5.46 ± 0.14 ^c^
45	C0	0.89 ± 0.02	3.03 ± 0.06 ^bc^	5.42 ± 0.05 ^bc^
	CWPI0	0.89 ± 0.13	2.82 ± 0.06 ^ac^	5.15 ± 0.08 ^ac^
	CWPI75	0.89 ± 0.10	2.99 ± 0.08 ^bc^	5.78 ± 0.12 ^abc^
	CWPIM	0.88 ± 0.13	2.45 ± 0.07 ^abc^	5.28 ± 0.17 ^c^
60	C0	0.91 ± 0.08	3.06 ± 0.01 ^bc^	5.25 ± 0.13 ^c^
	CWPI0	0.90 ± 0.04	3.19 ± 0.03 ^ac^	5.11 ± 0.16 ^c^
	CWPI75	0.91 ± 0.08	3.01 ± 0.05 ^bc^	5.29 ± 0.04 ^c^
	CWPIM	0.90 ± 0.08	2.68 ± 0.03 ^abc^	5.13 ± 0.14 ^c^

Statistical significance: one-way ANOVA—Dunnett’s test (^a^ control C0 vs. all samples, ^b^ sample CWPI0 vs. all samples, and ^c^ specific sample on day 1 vs. other days, *p* ≤ 0.05).

**Table 3:** Ash, total solids, and fat content (%) in control (C0) and WPI-coated cheese samples on days 1 and 60 of ripening.

Day	Sample	Ash (%)	Total Solids (%)	Fat (%)
1	C0	6.04 ± 0.12 ^b^	46.17 ± 0.10 ^b^	23.09 ± 0.18 ^b^
	CWPI0	5.34 ± 0.11 ^ac^	27.07 ± 0.17 ^a^	22.66 ± 0.08 ^ab^
	CWPI75	5.76 ± 0.20 ^bc^	46.36 ± 0.14 ^b^	21.88 ± 0.10 ^ab^
	CWPIM	5.55 ± 0.09 ^ac^	62.87 ± 0.13 ^ab^	21.75 ± 0.13 ^ab^
60	C0	2.20 ± 0.20	53.41 ± 0.07 ^bc^	25.24± 0.18 ^c^
	CWPI0	2.15 ± 0.19 ^c^	44.93 ± 0.10 ^ac^	25.54± 0.06 ^c^
	CWPI75	2.25 ± 0.05 ^c^	54.88 ± 0.08 ^abc^	25.29± 0.12 ^c^
	CWPIM	2.59 ± 0.14 ^abc^	48.32 ± 0.09 ^abc^	26.59± 0.17 ^abc^

Statistical significance: one-way ANOVA—Dunnett’s test (^a^ control C0 vs. all samples, ^b^ sample CWPI0 vs. all samples) and unpaired *t*-test (^c^ specific sample on day 1 vs. day 60 *p* ≤ 0.05).

**Table 4:** Total protein content, TCA-soluble nitrogen (TCA-SN), and water-soluble nitrogen (WSN) in control (C0) and WPI-coated cheese samples during ripening, as indicators of proteolysis.

Day	Sample	Total Protein (%)	TCA-SN (%)	WSN (%)
1	C0	18.39 ± 0.10	0.49 ± 0.13	1.68 ± 0.06 ^b^
	CWPI0	18.43 ± 0.16	0.70 ± 0.03	1.19 ± 0.13 ^a^
	CWPI75	19.19 ± 0.17 ^ab^	0.77 ± 0.14 ^a^	0.98 ± 0.15 ^a^
	CWPIM	19.87 ± 0.15 ^ab^	0.70 ± 0.02	0.98 ± 0.12 ^a^
15	C0	18.69 ± 0.08 ^c^	0.91 ± 0.04 ^c^	3.22 ± 0.18 ^bc^
	CWPI0	18.82 ± 0.14	0.84 ± 0.08	2.24 ± 0.04 ^ac^
	CWPI75	19.37 ± 0.16 ^abc^	0.56 ± 0.05 ^abc^	2.73 ± 0.02 ^abc^
	CWPIM	19.26 ± 0.15	0.42 ± 0.05	2.87 ± 0.10
30	C0	17.78 ± 0.06 ^bc^	1.82 ± 0.02 ^bc^	3.57 ± 0.07 ^bc^
	CWPI0	17.60 ± 0.03 ^ac^	1.54 ± 0.12 ^ac^	2.80 ± 0.04 ^ac^
	CWPI75	19.31 ± 0.09 ^ab^	1.75 ± 0.02 ^bc^	2.87 ± 0.02 ^ac^
	CWPIM	19.47 ± 0.06 ^abc^	1.75 ± 0.12 ^bc^	3.71 ± 0.05 ^abc^
60	C0	14.75 ± 0.07 ^bc^	1.61 ± 0.04 ^c^	4.48 ± 0.02 ^bc^
	CWPI0	17.44 ± 0.01 ^ac^	1.61 ± 0.05 ^c^	3.22 ± 0.16 ^ac^
	CWPI75	19.02 ± 0.12 ^ab^	2.17 ± 0.08 ^abc^	3.64 ± 0.03 ^abc^
	CWPIM	19.35 ± 0.05 ^abc^	2.17 ± 0.10 ^abc^	3.15 ± 0.09 ^ac^

Statistical significance: one-way ANOVA—Dunnett’s test (^a^ control C0 vs. all samples, ^b^ sample CWPI0 vs. all samples, and ^c^ specific sample on day 1 vs. other days, *p* ≤ 0.05).

**Table 5:** Color parameters (L*, a*, b*) and ΔE of WPI-coated cheese samples during ripening.

Day	Sample	L*	a*	b*	ΔE
1	C0	90.71 ^b^	−2.47 ^b^	14.61 ^b^	
	CWPI0	89.43 ^a^	−2.24	14.78 ^a^	1.31
	CWPI75	89.39 ^a^	−2.56	15.62 ^ab^	1.66
	CWPIM	91.01 ^ab^	−2.38	14.96 ^ab^	0.47
15	C0	91.38 ^bc^	−2.25 ^bc^	16.51 ^bc^	
	CWPI0	87.5 ^ac^	−1.56 ^ac^	19.4 ^ac^	4.89
	CWPI75	89.38 ^ab^	−2.03 ^abc^	14.55 ^abc^	2.81
	CWPIM	89.58 ^abc^	−2.12 ^abc^	17.41 ^abc^	2.02
30	C0	62.5 ^bc^	−0.79 ^bc^	13.59 ^bc^	
	CWPI0	59.85 ^ac^	−0.33 ^ac^	12.78 ^ac^	2.81
	CWPI75	74.33 ^abc^	−1.87 ^abc^	13.36 ^abc^	11.88
	CWPIM	75.27 ^abc^	−1.26 ^abc^	14.45 ^abc^	12.81
45	C0	80.76 ^bc^	−1.02 ^bc^	16.58 ^bc^	
	CWPI0	77.07 ^ac^	−1.63 ^ac^	27.48 ^ac^	11.52
	CWPI75	84.37 ^abc^	−2.46 ^ab^	22.23 ^abc^	6.86
	CWPIM	69.76 ^abc^	−1.76 ^abc^	28.25 ^abc^	16.05
60	C0	72.48 ^bc^	−0.79 ^bc^	10.67 ^bc^	
	CWPI0	67.34 ^ac^	0.39 ^ac^	9.18 ^ac^	5.48
	CWPI75	68.61 ^abc^	0.08 ^ac^	8.65 ^abc^	4.45
	CWPIM	73.56 ^abc^	−1.02 ^bc^	20.87 ^abc^	10.26

Statistical significance: one-way ANOVA—Dunnett’s test (^a^ control C0 vs. all samples, ^b^ sample CWPI0 vs. all samples, and ^c^ specific sample on day 1 vs. other days, *p* ≤ 0.05).

**Table 6:** Textural of control (C0) and WPI-coated cheese samples during 60 days of ripening.

Day	Sample	Hardness (N)	Gumminess (N)	Cohesiveness (mm)	Elasticity (mm)	Resilience
1	C0	7.75 ± 0.06	21.11 ± 0.05 ^b^	0.51 ± 0.13	−1.03 ± 0.18	0.47 ± 0.08
	CWPI0	7.48 ± 0.12	23.77 ± 0.06 ^a^	0.60 ± 0.02	−0.76 ± 0.01	0.57 ± 0.06
	CWPI75	8.07 ± 0.14 ^ab^	16.93 ± 0.07 ^ab^	0.51 ± 0.15	−0.52 ± 0.17 ^a^	0.46 ± 0.01
	CWPIM	10.54 ± 0.16 ^ab^	25.23 ± 0.15 ^ab^	0.62 ± 0.08	−0.97 ± 0.02	0.57 ± 0.19
15	C0	16.95 ± 0.11 ^bc^	4.39 ± 0.15 ^bc^	0.36 ± 0.10	−2.12 ± 0.15 ^c^	0.31 ± 0.16
	CWPI0	22.59 ± 0.09 ^ac^	7.72 ± 0.09 ^ac^	0.40 ± 0.19	−2.34 ± 0.07 ^c^	0.32 ± 0.17
	CWPI75	19.04 ± 0.13 ^abc^	2.99 ± 0.08 ^abc^	0.33 ± 0.02	−3.41 ± 0.19 ^abc^	0.27 ± 0.18
	CWPIM	25.65 ± 0.04 ^abc^	11.21 ± 0.06 ^abc^	0.42 ± 0.12	−1.32 ± 0.11 ^abc^	0.35 ± 0.16
30	C0	23.47 ± 0.17 ^bc^	3.74 ± 0.08 ^bc^	0.22 ± 0.09	−1.23 ± 0.04 ^b^	0.37 ± 0.17
	CWPI0	17.05 ± 0.02 ^ac^	4.71 ± 0.06 ^ac^	0.21 ± 0.18 ^c^	−1.54 ± 0.14 ^ac^	0.30 ± 0.04
	CWPI75	23.99 ± 0.13 ^abc^	6.00 ± 0.18 ^abc^	0.32 ± 0.20	−3.09 ± 0.12 ^abc^	0.31 ± 0.06
	CWPIM	24.45 ± 0.03 ^abc^	8.76 ± 0.20 ^abc^	0.34 ± 0.08 ^c^	−3.46 ± 0.15 ^abc^	0.25 ± 0.12
45	C0	22.20 ± 0.16 ^bc^	6.21 ± 0.15 ^bc^	0.26 ± 0.18	−1.18 ± 0.11 ^b^	0.39 ± 0.14
	CWPI0	33.07 ± 0.06 ^ac^	5.72 ± 0.09 ^ac^	0.34 ± 0.06	−3.10 ± 0.15 ^ac^	0.25 ± 0.15 ^c^
	CWPI75	23.27 ± 0.09 ^abc^	8.90 ± 0.06 ^abc^	0.37 ± 0.17	−3.35 ± 0.11 ^ac^	0.30 ± 0.08
	CWPIM	31.66 ± 0.01 ^abc^	9.65 ± 0.12 ^abc^	0.39 ± 0.14	−2.90 ± 0.10 ^ac^	0.28 ± 0.14
60	C0	41.46 ± 0.14 ^bc^	4.99 ± 0.20 ^bc^	0.22 ± 0.16	−5.59 ± 0.01 ^bc^	0.15 ± 0.19
	CWPI0	39.63 ± 0.05 ^ac^	9.14 ± 0.08 ^ac^	0.28 ± 0.12 ^c^	−4.56 ± 0.16 ^ac^	0.21 ± 0.17 ^c^
	CWPI75	33.07 ± 0.16 ^abc^	6.29 ± 0.08 ^abc^	0.27 ± 0.04	−3.17 ± 0.06 ^abc^	0.20 ± 0.05 ^c^
	CWPIM	40.65 ± 0.14 ^abc^	9.63 ± 0.11 ^abc^	0.30 ± 0.08 ^c^	−3.11 ± 0.06 ^abc^	0.23 ± 0.14

Statistical significance: one-way ANOVA—Dunnett’s test (^a^ control C0 vs. all samples, ^b^ sample CWPI0 vs. all samples, and ^c^ specific sample on day 1 vs. other days, *p* ≤ 0.05).

## Data Availability

The original contributions presented in the study are included in the article/Supplementary Material, further inquiries can be directed to the corresponding author.

## Supplementary Materials

The following supporting information can be downloaded at: https://www.mdpi.com/article/10.3390/foods14091496/s1, Figure S1: Inhibition zones of the OLE (20 µL in triplicate; inhibition zones 12 ± 0 mm) against *S. aureus* (K—kanamycin 50 µg disk—positive control); Figure S2: *S. aureus* density in 10 µL of a serial 10-fold dilution after 18 hours of incubation without extract (A—control), in the presence of 50 % extract (B) and in the presence of 10 µg mL^−1^ kanamycin (C). Log CFU mL^−1^: A—9.48; B—6.22; C—6.30. The MIC value of 50 % extract and 10 µg mL^−1^ kanamycin was determined by staining with resazurin; Figure S3: Inhibition zones of WPI-based coatings A (WPI0), B (WPI75) and C (WPIM) against *S. aureus* (K—kanamycin 50 µg disk—positive control).
