# Supplementary material for: Bioactive Properties of the Microwave-Assisted Olive Leaf Extract and Its Incorporation into a Whey Protein Isolate Coating of Semi-Hard Cheese
Source: Foods. 2025 Apr 25;14(9):1496. doi: 10.3390/foods14091496 (PMC12071697; doi:10.3390/foods14091496)
Supplement: Supplementary file 1 [file foods-14-01496-s001.zip › foods-3592189-supplementary.pdf]

Supplementary

Bioactive Properties of the Microwave-Assisted Olive Leaf Extract and Its Incorporation into a Whey Protein Isolate Coating of Semi-Hard Cheese

Elizabeta Zandona [1], Maja Vukelić [2], Karla Hanousek Čiča [2], Antonio Zandona [3], Jasna Mrvčić [2], Maja Katalinić [3], Ines Cindrić [1], Almir Abdurramani [4] and Irena Barukčić Jurina [2,*]

[1] Department of Food Technology, Karlovac University of Applied Sciences, Trg J.J. Strossmayera 9, 47000 Karlovac, Croatia; elizabeta.zandona@vuka.hr (E.Z.); ines.cindric@vuka.hr (I.C.)
[2] Department of Food Engineering, Faculty of Food Technology and Biotechnology, University of Zagreb, Pierottijeva 6, 10000 Zagreb, Croatia; majavukelic79@gmail.com (M.V.); khanousekcica@pbf.hr (K.H.Č.); jmrvcic@pbf.hr (J.M.)
[3] Division of Toxicology, Institute for Medical Research and Occupational Health, Ksaverska Cesta 2, 10000 Zagreb, Croatia; azandona@imi.hr (A.Z.); mkatalinic@imi.hr (M.K.)
[4] Faculty of Food Technology, Josip Juraj Strossmayer University of Osijek, Franje Kuhača 18, 31000 Osijek, Croatia; abdurramanialmir@gmail.com
* Correspondence: irena.barukcic@pbf.unizg.hr; Tel.: +385-1-4605-017

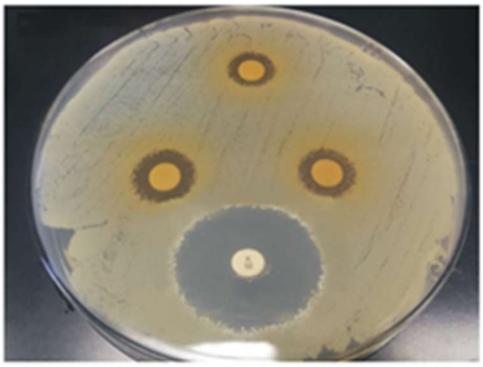

Figure S1. Inhibition zones of the OLE (20 μL in triplicate; inhibition zones 12±0 mm) against *S. aureus* (K - kanamycin 50 μg disk - positive control).

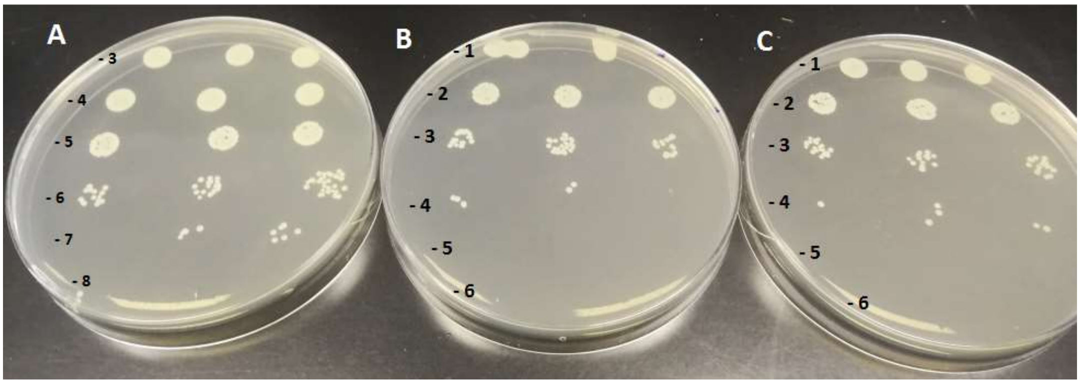

Figure S2. *S. aureus* density in 10 μL of a serial 10-fold dilution after 18 hours of incubation without extract (A - control), in the presence of 50 % extract (B) and in the presence of 10 μg mL^{-1} kanamycin (C). Log CFU mL^{-1}: A - 9.48; B - 6.22; C - 6.30. The MIC value of 50 % extract and 10 μg mL^{-1} kanamycin was determined by staining with resazurin.

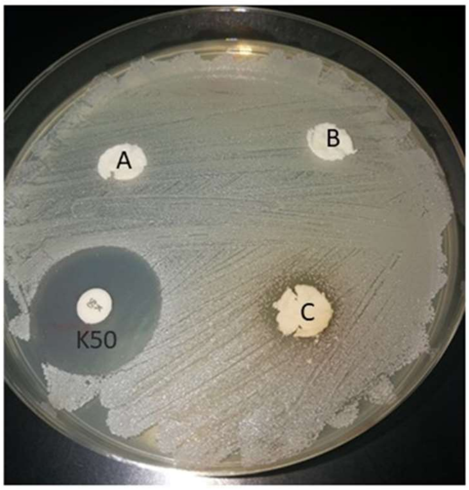

Figure S3. Inhibition zones of WPI-based coatings A (WPI0), B (WPI75) and C (WPIM) against *S. aureus* (K - kanamycin 50 μg disk - positive control).
